# Massive Uterine Leiomyoma in a Patient with Friedreich's Ataxia: Is There a Possible Association?

**DOI:** 10.1155/2011/648217

**Published:** 2011-08-14

**Authors:** Evangelos P. Misiakos, Elli Siama, Dimitrios Schizas, Constantinos Petropoulos, Nikos Zavras, Nikos Economopoulos, Alexandros Charalabopoulos, Anastasios Macheras

**Affiliations:** ^1^3rd Department of Surgery, University of Athens School of Medicine, Attikon University Hospital, Haidari, Athens 12462, Greece; ^2^2nd Department of Radiology, University of Athens School of Medicine, Attikon University Hospital, Haidari, Athens 12462, Greece; ^3^University of Athens School of Medicine, Attikon University Hospital, Haidari, Athens 12462, Greece; ^4^Department of Pediatric Surgery, University of Athens School of Medicine, Attikon University Hospital, Haidari, Athens 12462, Greece

## Abstract

A possible association between Friedreich's ataxia (FA) and neoplastic development has been recognized. FA patients have low frataxin levels and insufficient response to oxidative stress. In these patients fibroblasts are characterized by a high rate of mutations. Herein, a case of a 39-year-old woman with FA tetraplegia, who was admitted in our department with intestinal obstruction due to a huge uterine tumor, is described. An abdominal CT revealed a huge intra-abdominal mass originating from the right cornu of the uterus. Tumor excision and adhesionlysis were performed. The histological examination of the tumor revealed a leiomyoma. FA patients seem to present with a variety of neoplasms uncommon for their young age. This is the first report of a leiomyoma originating from the genital system in a female patient with FA tetraplegia. Therefore it is important to identify neoplasms at an early stage in patients with FA and start immediate therapy.

## 1. Introduction

Friedreich's Ataxia (FA) is an autosomal recessive disease of the cerebellum, spinal cord, and peripheral nerves described by Friedreich in 1863 [1]. It is characterized pathologically by degeneration of the spinal and cerebellar reflexes and clinically by ataxic gait, dysarthria, loss of reflexes, and heart disease [2–4]. Although incidences of coexisting tumors of the connective tissue have been reported in this group of patients and the question of a correlation of the condition with malignancies in general has been raised [3], a possible association between neoplasias of the connective tissue (benign or malignant) and FA has not been previously discussed. Herein, we present a case of a 39-year-old patient with a history of FA and a huge uterine leiomyoma, who was admitted with symptoms of acute intestinal obstruction. This case illustrates the importance of reporting such cases and of further investigation in this field. Moreover, it stresses the clinicians' attention in becoming more sensitive on early diagnosis and treatment of such neoplasias in this group of patients.

## 2. Case Presentation

A 39-year-old white female patient was admitted to the emergency department of our hospital with symptoms of acute intestinal obstruction, first noted 48 hours before (colicky abdominal pain, vomiting, and abdominal distention). The patient was tetraplegic due to Friedreich's disease diagnosed 25 years ago and was hospitalized permanently in a center for chronic disorders.

Upon physical examination, a prominent abdominal mass distorting her trunk shape was noted. In palpation, the abdomen had considerable distension with diffuse tenderness and moderate rebound tenderness. Despite the distension in the upper abdomen, a huge tumor could easily be palpated occupying mainly the lower abdomen.

The neurological examination revealed good responsiveness of the patient, quadriplegia, loss of tendon reflexes, and positive Babinski sign bilaterally. She was then referred for cardiac evaluation, where mitral insufficiency and left ventricular hypertrophy were found. 

The white blood cell count was 18,100/*μ*L with 79.1% neutrophils, which was further elevated the second day; urea was found 51 mg/dL and *γ*GT 54 mU/mL. The rest of the blood tests were normal.

**Figure 1:**
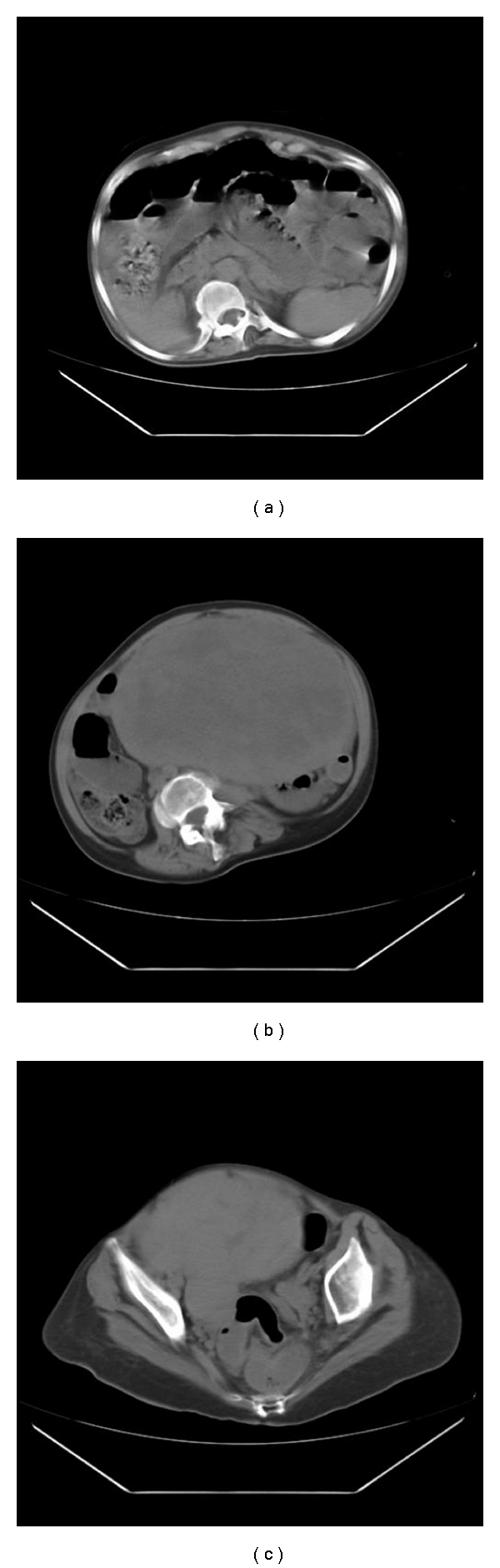
(a–c) MRI images indicating a huge mass occupying the greatest part of the abdominal cavity compressing the intestinal loops.

The most possible diagnosis was intestinal obstruction probably due to the tumor. An abdominal computed tomography was performed, which revealed a huge intra-abdominal mass originating most possibly from the right cornu of the uterus (Figures 1(a), 1(b), 1(c)). 

An emergency exploratory laparotomy followed with a midline incision. 

**Figure 2:**
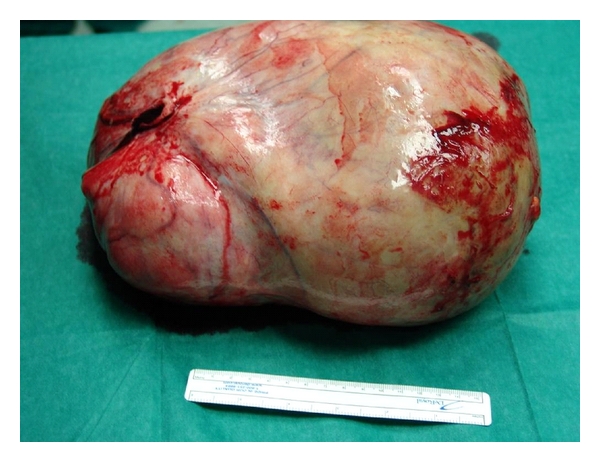
The huge compact tumor after being removed from the abdominal cavity.

After the peritoneal cavity was opened an enormous circumscribed mass measuring 29.5 × 22 × 12 cm was revealed displacing the bowel to the abdominal periphery. The mass originated from the right cornu of the uterus. After lysis of the adhesions between the tumor and the greater omentum, the tumor was excised (Figure 2) and the right uterine cornu was ligated with a nonabsorbable suture. Inspection of the small intestine revealed a stenotic area in its central portion due to an adhesion with the omentum. The adhesion was taken down and careful hemostasis followed. Finally the abdominal wound was closed in the ordinary fashion.

The tumor, as well as the peritoneal washings, was sent for histological and cytological assessment, which did not reveal any evidence of malignancy. Histological assessment of the specimen revealed a uterine leiomyoma with increased mitotic activity. The postoperative course was uneventful, and the patient had no further complications. At present, two years after surgery the patient fares well and lives in a center for chronic disorders.

## 3. Discussion

 FA is an autosomal recessive spinocerebellar neurodegenerative disease with a wide range of clinical signs and symptoms, the onset of which in the majority of the cases occurs during adolescence. Although FA is a rare condition, it is the most common hereditary ataxia. The disease results in a relatively low life expectancy, and therefore the survival beyond the forth decade is unusual [2].

 The neurological findings of the disease include limb and truncal ataxia, dysarthria, loss of lower-extremity tendon reflexes, and progressive corticospinal tract disorder as well as loss of vibratory and proprioceptive sensation. The disease has also nonneurological manifestations such as kyphoscoliosis, pes cavus, diabetes mellitus with insulin resistance and heart disease, atrial fibrillation with resultant tachycardia, and cardiomyopathy [3, 5].

 FA is caused by a pronounced lack of fraxatin, a mitochondrial protein of not well-understood function [2]. The underlying molecular defect leading to the clinical features of FA is the mutation of the frataxin gene located in chromosome 9q13. The result of this mutation is an expansion of repeated GAA triplets in intron I, the length of which is strongly associated with the severity of the clinical appearance [6]. 

 Frataxin is a mitochondrial protein found predominately in the brain, heart, and pancreas. Although the main function of frataxin is still a matter of discussion [7], some evidence suggests that this protein directs the intramitochondrial synthesis of Fe/S clusters [8–10] and is undoubtedly involved in the control of oxidative metabolism by reinforcing the ATP synthesis possibly due to a direct interaction with the respiratory chain [11, 12]. Frataxin is overexpressed in fibroblasts. Moreover, disruption of the frataxin homologue in yeast has been proven to increase sensitivity to oxidants and promote oxidative damage to both nuclear and mitochondrial DNA [13, 14]. Lack of hepatic frataxin expression causes liver tumor growth in mice following impaired mitochondrial function and increased ROS formation. Hence, frataxin might be considered a mitochondrial tumor suppressor protein located upstream of established stress kinases in mammals, such as p38 MAP kinase [15]. Moreover, Schulz et al. [16] have shown that induction of oxidative metabolism by mitochondrial frataxin suppresses malignant growth in vitro and in vivo, due to the fact that mitochondrial iron detoxification is a primary function of frataxin. Frataxin seems to limit oxidative damage and preserve cell longevity [17].

 Fraxatin not only protects tumor cells against oxidative stress and apoptosis but also acts as a tumor suppressor. Fraxatin expression is upregulated in several tumor cell lines in response to hypoxic stress, a condition often associated with tumor progression. The ability of tumor cells to maintain a balance between adaptation to hypoxia and cell death is mediated by hypoxia-inducible factors (HIFs), which regulate the expression of hypoxia-responsive genes [18]. Therefore fraxatin upregulation in response to hypoxia depends on HIF expression, and this modulates activation of the tumor suppressor gene p53, while fraxatin dampens oxidative stress. Overall fraxatin participates in the hypoxia-induced stress response in tumors, and modulation of its expression could have an important role in neoplastic cell survival and tumor progression [18]. 

 FA patients have low frataxin levels and insufficient response to oxidative stress [19]. In this subgroup of patients, fibroblasts are characterized by a particular sensitivity to ionizing radiation and a high rate of mutations [20]. The aforementioned facts could support a hypothesis of an association between FA and tumor development, thus of the connective tissue. We have presented a noteworthy case of a 39-year-old female patient with history of Friedreich's disease and a uterine leiomyoma, indicating the coexistence of FA and an early onset neoplasia of the connective tissue. 

 Recently, two enzymes involved in the tricarboxylic acid (TCA) cycle, fumarate hydratase (FH), and succinate dehydrogenase (SDH), which also plays a role in oxidative phosphorylation, have been shown to be tumor suppressors. Indeed germline mutations of FH predispose individuals to the development of uterine or cutaneous leiomyomas, whereas mutations in SDH cause hereditary paragangliomas and pheochromocytomas [21]. On the other hand, FH deficiency has been identified in a few cases of encephalomyopathy, whereas a generalized deficiency of iron-sulfur proteins, including SDH, causes Friedreich ataxia [22]. These observations may offer strong evidence in the genetic association between FA and leiomyoma development. 

 Although tumor development is not a typical manifestation of the disease, these patients seem to present with a variety of neoplasms uncommon for their age. To the best of our knowledge, six cases of neoplasias with FA have been described in the literature, but none of them was associated with leiomyoma. In summary, in 1986, Barr et al. [23] presented the case of a small primary ganglioneuroblastoma and FA. In 1996 Ackroyd et al. [24] reported the case of two siblings with FA who both developed a signet ring cell adenocarcinoma of the stomach stating the assumption of the existence of unidentified aberrant gene. In 1999, De Pas et al. [5] described a case of coexisting FA and lymphoblastic lymphoma, while in 2001 the case of two sisters with FA and breast cancer was added in world literature by Kidd et al. [25]. The list of cases is further grown in 2004, when Shah et al. [26] published an additional case of neurofibromatosis type I and FA similar to the one described in 1999 in a Nigerian family [27].

 Although the question of a possible association between FA and malignancy in general was raised by Kidd and his colleagues [24], it has not been clarified yet. We support the hypothesis of an association between FA and neoplasias of the connective tissue (benign or malignant), although such a causal relationship would be nearly impossible to demonstrate statistically due to the low number of cases. This could be explained by an underreporting of FA and such tumors occurring together since the neoplasias of the connective tissue are very common in the general population. We know, for example, that leiomyoma is a very common condition in the general population but we do not know how common it is in patients with FA and if there is a statistically important difference. Moreover, the patients with early onset of FA die at a young age mostly because of cardiomyopathy and consequently they do not live long enough to develop such tumors.

 However, it is important to identify tumor development at an early stage in patients with FA and start immediate therapy; therefore the clinician should be sensitive enough to detect them.

##  Conflict of Interests

The authors declare that there is no conflict of interests.
